# EFFECT OF PASTEURIZATION ON THE ANTIOXIDANT AND OXIDANT PROPERTIES OF HUMAN MILK

**DOI:** 10.1590/1984-0462/2021/39/2019165

**Published:** 2020-08-10

**Authors:** Mariane Fioroti Lorençoni, Racire Sampaio Silva, Romildo Azevedo, Marcio Fronza

**Affiliations:** a Universidade Vila Velha, Vila Velha, ES, Brazil.

**Keywords:** Antioxidants, Milk banks, Oxidative stress, Milk, human, Pasteurization, Antioxidantes, Bancos de leite, Estresse oxidativo, Leite humano, Pasteurização

## Abstract

**Objective:**

To evaluate the effect of pasteurization on antioxidant and oxidant properties of human milk.

**Methods:**

42 samples of milk before and after pasteurisation were used to evaluate the antioxidant activity by the ferric reducing capacity and by scavenging the 2,2’-azino-bis 3-ethylbenzthiazoline-6-sulfonic acid radical. Lipid peroxidation was estimated by the concentration of malondialdehyde product using the thiobarbituric acid reactive substances assay and by the evaluation of advanced oxidation protein products.

**Results:**

No significant difference was observed in fresh human milk and after pasteurization in relation to antioxidant properties determined by the ferric reducing capacity (50.0±3.4% and 48.8±3.0%, respectively) and by scavenging the 2,2’-azino-bis 3-ethylbenzthiazoline-6-sulfonic acid radical (28.9±1.5% and 31.2±1.3%, respectively). The results of malondialdehyde (62.6±4.1 and 64.3±3.6 µM/mg) and protein oxidation products (59.4±3.4 and 54.2±3.8 µM/L) of fresh and pasteurized milk, respectively, did not exhibited any significant difference.

**Conclusions:**

This data showed that human milk has an important antioxidant activity and that the pasteurizing process does not influence the antioxidant capacity, avoiding the peroxidation of breast milk lipids and the formation of advanced protein oxidation products.

## RESUMO

**Objetivo:**

Avaliar o efeito da pasteurização nas propriedades antioxidantes e oxidantes do leite humano.

**Métodos:**

Foram utilizadas 42 amostras de leite cru e após a pasteurização, para avaliação da atividade antioxidante pelos métodos da capacidade redutora do ferro e sequestro dos radicais derivados do ácido 2,2’-azino-bis (3-etilbenzotiazolina-6-sulfônico). A peroxidação lipídica (PL) foi estimada pela determinação das substâncias reativas ao ácido tiobarbitúrico e pela avaliação dos produtos proteicos de oxidação avançada.

**Resultados:**

Não se observou diferença significante no leite humano cru nem após a pasteurização em relação às propriedades antioxidantes determinadas pelo método da redução do ferro (50,0±3,4% e 48,8±3,0%, respectivamente) e pelo sequestro dos radicais derivados do ácido 2,2’-azino-bis (3-etilbenzotiazolina-6-sulfônico) (28,9±1,5% e 31,2±1,3%, respectivamente). Os resultados médios de malondialdeído [MDA] (62,6±4,1 e 64,3±3,6 µM/mg) e produtos de oxidação proteica (59,4±3,4 e 54,2±3,8 µM/L) entre os grupos leite fresco e leite pasteurizado, respectivamente, não evidenciaram diferença significante.

**Conclusões:**

Os dados demonstraram que o leite humano possui importante atividade antioxidante e que o processo de pasteurização não interfere nessa propriedade, evitando assim a peroxidação dos lipídios e a formação de produtos avançados de oxidação proteica.

## INTRODUCTION

The importance of human milk in feeding infants is undeniable. This is due to the fact that breast milk is, arguably, the most complete food that the baby can receive, as it provides all the nutrients, vitamins and minerals that he needs for growth in the first months. According to the lactation phase, it is called: colostrum until the sixth day, transition milk until the 14th day and mature milk after 15 days. In addition, the content changes during feeding and if the baby is full term or premature.^1^ ^,^ ^2^

Human milk is one of the most efficient ways to attend to the nutritional, immunological and psychological aspects of children in their first year of life. The World Health Organization (WHO) recommends exclusive breastfeeding for 4-6 months and supplemented for up to two years or more, being safe to be offered exclusively and on demand, without restrictions on time or quantity. However, in some cases, this exclusive feeding directly from the breast is not always available, and using certified milk banks or commercially available milk formulas is required.^2^ ^,^ ^3^

It should be noted that the use of milk formula in one hospitalization interferes with the duration of exclusive breastfeeding, promotes increased oxidative stress (OS), in addition to modifying the benefits achieved by the intestinal microbiota developed by exclusive breastfeeding.^4^ ^,^ ^5^ In the absence of direct feeding from the breast, the recommendation by the principal competent entities, including the American Academy of Pediatrics, so that the benefits related to the use of human milk are not lost, is the use of raw human milk from the mother herself for her child or pasteurized milk found in milk banks.^6^ ^,^ ^7^ ^,^ ^8^ ^,^ ^9^

Studies addressing the influence of pasteurization on the maintenance of biological factors have been carried out. Traditional pasteurization (62.5 °C, for 30 min) maintains the protein profile of human milk without major changes.^8^ As for the antioxidant properties, pasteurization caused a significant drop in the activity of two antioxidant enzymes - superoxide dismutase and glutathione peroxidase -, while the freezing/storage of raw milk only affected superoxide dismutase.^7^ It is known today that the use of breast milk in infant feeding can reduce the risk of obesity, even if it is produced by obese mothers.^10^ Studies concerning OS are correlated with the imbalance between reactive oxygen species (ROSs) and reactive nitrogen species (NREs) and with the efficiency of the antioxidant defense system. Breastfed children have been shown to have a more efficient antioxidant barrier when compared to formula-fed children.^7^ ^,^ ^11^ ^,^ ^12^ Fats are the greatest source of energy in human milk and are so important that studies are being directed in such a way that milk formulas seek to mimic their lipid profile.^13^ ^,^ ^14^ The main fatty acids found in human milk are restricted to those with chains of 12 to 18 carbons. Among them, linoleic and linolenic acids stand out, which are considered essential fatty acids and the precursors of long-chain polyunsaturated fatty acids (LCPUFA) - arachidonic acid and docosahexaenoic acid. The preterm newborn, especially the one with very low weight, has limited capacity to synthesize LCPUFA through its precursors, which shows the importance of its supply for human milk.^1^ ^,^ ^13^ ^,^ ^15^

LCPUFA are considered fundamental for brain growth and development, as well as for the cognitive development of the newborn.^13^ ^,^ ^14^ ^,^ ^15^ In this sense, the potential role of SO is questioned, resulting from the imbalance between pro-oxidizing agents (free radicals) and the antioxidant defense mechanisms of the puerperal organism and breast milk itself, the only source of LCPUFA for the newborn.^11^ ^,^ ^12^ ^,^ ^13^ ^,^ ^14^ ^,^ ^15^ ^,^ ^16^ Protein oxidation products (AOPP) have also been understood as new markers of oxidation and damage to proteins and can be used to estimate protein oxidative damage.^12^ ^,^ ^13^ ^,^ ^14^ ^,^ ^15^ ^,^ ^16^ ^,^ ^17^ Malondialdehyde (MDA) is one of the final products of lipid peroxidation (LP) and, being a stable product, it can be used as a cumulative measure of this process.^16^ Therefore, this study aimed to evaluate the antioxidant and oxidant profile of fresh human milk and after the pasteurization process.

## METHOD

42 samples of fresh and pasteurized milk were collected in March and April 2018 and kindly provided by the Human Milk Bank of the Hospital Estadual Infantil e Maternidade Dr. Al zir Bernardino Alves (HEIMABA), in Vila Velha, Espírito Santo, Brazil. The sample size was calculated considering the design of the total antioxidant activity. To estimate variability for this characteristic, it was based on Nogueira et al., who obtained a standard deviation of approximately 10 microMol for the total antioxidant activity test (ABTS).^18^ For dimensioning, a significance level of 5%, power of 80%, magnitude of effect of 9 microMol and paired Student’s *t-*test as an inferential test, reaching the minimum size of 18 samples when applying the formula.

After collection, 25 mL of each sample was transported in an isothermal box at a temperature of 3 to 4 °C to the Vila Velha University (UVV) laboratory, where it was immediately frozen at -16 °C, the usual temperature for preserving milk in human milk banks. After a period of 15 days, all samples were defrosted at room temperature and gently homogenised for analysis. The study was approved by the UVV Human Research Ethics Committee under number 1804463.

The pasteurization process was carried out as recommended by the Brazilian Human Milk Bank Network.^9^ To inactivate pathogenic microorganisms and saprophytic microbiota, the milk was heated to 62.5 °C for 30 minutes. During the heating time, it was moderately stirred, to avoid adhesions to the walls of the container, to promote uniform heating of all its particles and, at the same time, to avoid the formation of foam. After cooling, it was stored at -16 °C.

The reagents TPTZ - 2.4.6-tri (2-pyridyl) -1.3.5-triazin, potassium persulfate, sodium acetate tri -hidrate, glacial acetic acid, concentrated hydrochloric acid, ferric chloride 2 2- the zino-bis (3-ethylbenzthiazoline sulfonic acid-6) (ABTS), thiobarbituric acid - TBA (reactive substances to thiobarbituric acid - TBARS), ultrapure acetic acid, tocopherol, human albumin and chloramine T were purchased from Sigma-Aldrich^®^ Chemical Co. (St. Louis, MO, Estados Unidos). All other reagents and solvents used were obtained commercially and had an analytical grade.

The antioxidant activity was determined by the modified *ferric reducing antioxidant power* (FRAP) method, as an alternative for the analysis of biological fluids.^16^ In this method, the ferric-tripyridyltriazine complex (Fe III-TPZ) is reduced to the ferrous complex (Fe II-TPZ), in the presence of an antioxidant under acidic conditions. The complex formed was determined at 595 nm. The experiments were carried out in triplicate and the data expressed as a percentage of radical reduction, being representative of at least two independent experiments.

The antioxidant activity of milk samples was also determined by the free radical scavenging method ABTS (Sigma-Aldrich^®^, St. Louis, MO, Estados Unidos).^16^ The experiments were performed in triplicate and the data expressed as a percentage of radical reduction, being representative of at least two independent experiments.

The quantification of total proteins in the milk samples was determined by the Bradford colorimetric method (Sigma-Aldrich^®^, St. Louis, MO, United States),^19^ and the total protein content in breast milk was calculated through analysis of linear regression using the straight line equation obtained by constructing the standard albumin curve (Sigma-Aldrich^®^, St. Louis, MO, United States). The results were expressed in mg/ml.

The content of LP related to the OS was evaluated by testing the thiobarbituric acid reactive substances as described by Ansarin et al., with modifications.^20^ For every 50 µL of milk (previously diluted 1:20 with deionized water), 200 µL of thiobarbituric acid (Sigma Aldrich^®^, St. Louis, MO, United States) was added, and the sample was incubated at 100 °C for 2h30 and shaken, to avoid the crystal formation. Then, 200 µL of each sample was transferred to a 96-well plate, and the absorbances were read at 532 nm. The concentrations were obtained in nmol of MDA (Sigma-Aldrich^®^, St. Louis, MO, United States) and later normalized with the protein content measured in the same samples by the Bradford method. The final result was expressed in nmol of MDA/mg of proteins.

The analysis of the evaluation content of the AOPP was carried out according to Talukder et al.,^12^ modified, in comparison to the reactions of chlorinated oxidizing agents (chloramines). The samples were diluted in phosphate buffered saline (in the proportion 1:30 and/or 1:50), and then 10 µL of potassium iodide (KI) (1.16 M) and 20 µL of acetic acid were added (Sigma-Aldrich^®^, St. Louis, MO, United States). The plate was shaken for six minutes, and the absorbance of the reaction was immediately read at 340 nm. The AOPP content was calculated based on a standard chloramine T curve. The results were expressed in µmol of chloramine T/mg protein equivalents.

Statistical analyzes were performed using the GraphPad software (San Diego, CA, United States) and the data expressed as mean±standard deviation (SD). Statistical differences between groups were determined using Student’s t test, and p values <0.05 were considered significant.

## RESULTS

**Figure 1:**
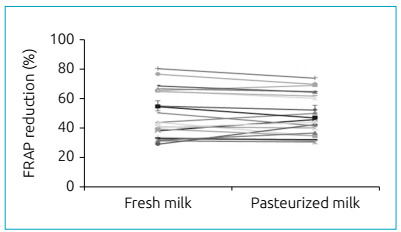
Antioxidant activity of fresh human breast milk and after the pasteurization process determined by the iron reducing capacity (FRAP). Mean values ± standard deviation in percentage of reduction of the FRAP radical obtained with fresh milk and pasteurized milk (n=21). There was no statistical difference between groups for p>0.05.

The determination of the antioxidant activity of breast milk allows the global characterization of its value, enabling the minimization of OS in newborns. Several techniques have been used to determine the antioxidant activity *in vitro* by means of biological fluids, highlighting the FRAP technique (iron reducing capacity), which determines the antioxidant effect of milk, via the evaluation of the reduction of the Fe^3+^ complex - TPTZ (ferritripyridyltriazine) to ferrous-tripiri-diltriazine (Fe^2+^-TPTZ).^16^ In the analysis of fresh human milk samples using the FRAP assay, an average iron reducing activity of 50% was observed. For samples of pasteurized human milk, an average reducing activity of 48.8% was found, with no significant differences between groups (Figure 1).

**Figure 2:**
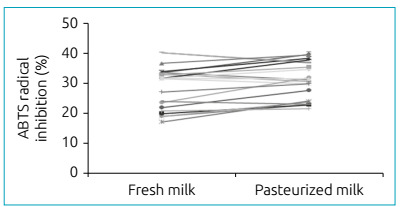
Antioxidant activity of fresh human breast milk and after the pasteurization process determined by the method of total antioxidant activity test. Mean values ± standard deviation in percentage of inhibition of the free radical ABTS^• +^ obtained with fresh milk and pasteurized milk (n=21). There was no statistically significant difference between groups (p>0.05).

Another test widely used to assess antioxidant activity in biological fluids is the ABTS test, which monitors the decay of the ABTS radical cation produced by the oxidation of ABTS^•+^ when a sample containing antioxidants is added.^16^ Using the ABTS technique, as well as seen in the FRAP test, there was no significant difference between the groups, as shown in Figure 2.

**Figure 3:**
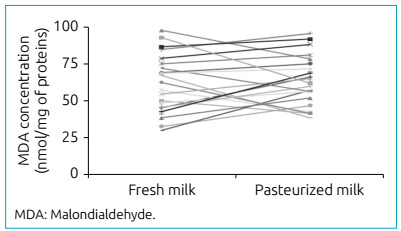
Malondialdehyde concentration in fresh human breast milk and after the pasteurization process determined by the method of substances reactive to thiobarbituric acid. Mean values ± standard deviation in nmol of MDA/mg of proteins obtained with fresh milk and pasteurized milk (n=21). There was no statistically significant difference between groups (p>0.05).

The LP was determined by the TBARS quantification method. This test is widely used to estimate the peroxidation of lipids in membranes and in biological systems such as human milk.^21^ ^,^ ^22^ The results obtained demonstrated that the LP determined in fresh milk (62.6±4.1 nM/mg of proteins) and pasteurized (64.3±3.6 nM/mg of proteins) did not present significant difference in the concentration of MDA between the groups (Figure 3).

**Figure 4:**
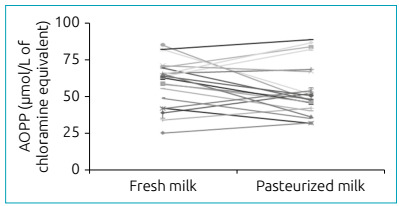
Evaluation of protein products of advanced oxidation of fresh human breast milk and after the pasteurization process. Mean values ± standard deviation in µmol/L of chloramine equivalents T/mg of proteins obtained with fresh milk and pasteurized milk (n=21). There was no statistically significant difference between groups (p>0.05).

According to what was observed in Figure 4, there was no significant difference between the fresh milk samples and after the pasteurization process regarding the AOPP levels (59.4±3.4 *versus* 54.2± 3.8 µM/L, respectively).

## DISCUSSION

Our results of the antioxidant tests showed that both fresh and pasteurized milk showed important antioxidant activity. Several studies corroborate the findings in the present study, confirming the antioxidant properties of fresh milk, breast milk of mothers of preterm and full-term babies.^7^ ^,^ ^12^ ^,^ ^23^ Research on the maintenance of the antioxidant properties of pasteurized milk is controversial. Bertino et al.^6^ describe that there are no significant changes in the antioxidant profile after the pasteurization process. On the other hand, a negative effect of the pasteurization process followed by the freezing of breast milk was observed on the content of total phenolics, accompanied by a consequent decrease in the total antioxidant capacity in the first seven days of storage.^18^

Breast milk contains numerous antioxidant peptide precursor proteins, very important for the control of EO, which occurs even in normal situations and is aggravated in situations of stress, low birth weight and in cases of admission to the Neonatal Intensive Care Unit (NICU), in which the use of pasteurized breast milk is necessary, when expressed breast milk is not sufficient. Premature infants are specifically sensitive to free radicals because of some peculiar situations, such as hypoxic-hyperoxic challenge, infections, deficiency in antioxidant defense and high levels of free iron.^21^ ^,^ ^24^ Hence the great importance of offering a food that enhances the scavenging capacity of free radicals, especially for these patients in a special way.

 The antioxidant action of milk is also considered of paramount importance for the prevention of LP and the scavenging capacity of free radicals. In this study, neither fresh milk nor pasteurized milk showed significant levels of LP. Thus, it is inferred that the antioxidant content of milk may have contributed to minimize or decrease lipid and protein degradation. Corroborating with the findings of the present study, Silvestre et al.^23^ noted that the concentration of MDA in the milk samples follows a normal distribution in all groups and that the values obtained were similar in the samples of fresh and pasteurized milk. In another study, carried out by Terek et al. TBARS also showed similar values between groups when analyzing the milk of mothers of preterm and full-term babies were analyzed.

AOPP can be considered as protein oxidation markers generated by the reaction between proteins and chlorinated oxidants derived mainly from myeloperoxidase by activated neutrophils.^12^ ^,^ ^16^ ^,^ ^17^ In this context, it can be speculated that the pasteurization process is an important step in the process of preserving the protein and lipid integrity of breast milk, due to the inactivation of polymorphonuclear neutrophil leukocytes present in it. Regarding the effects of pasteurization on the biological components of human milk, a possible explanation for the significant variability in the data reported in the scientific literature is the heterogeneity of the test and study protocols (for example, in terms of sample origin, storage conditions or methods of analysis). Another important source of variability is represented by the fact that pasteurization of donor milk is simulated in small rates in some studies, instead of being carried out following protocols implemented by the Human Milk Bank.^8^ ^,^ ^9^

In general, pasteurized milk remains close to fresh in the final composition, according to the practice used and recommended by milk banks, since pasteurization is necessary to protect the newborn that will receive the milk. The vast majority of newborns who receive this pasteurized milk in Brazil are made up of babies admitted to NICUs, who are already in a situation of fragility and stress. Despite recent studies demonstrating new pasteurization techniques, the method of choice and recommended by the Ministry of Health of Brazil for the Brazilian Network of Milk Banks is still the traditional one.^25^ ^,^ ^26^ Corroborating the results presented in this study, Elisia and Kitts also confirmed that the traditional process of pasteurization of breast milk did not significantly affect the antioxidant capacity of human milk, nor the lipid oxidation in human milk, assessed by determining the average concentration of MDA in samples of raw human milk and after pasteurization.^2^ ^,^ ^27^

Our study has two limitations to highlight. First, it was not one to research designed to evaluate the effects of pasteurization in different types of breast milk, colostrum, transitional and mature. Secondly, when assessing the effects of pasteurization on the anti- oxidant and pro-oxidant activity, only the freezing time of less than 15 days was taken into account.

Based on the results presented here, we can conclude that the conventional pasteurization process of human milk, as recommended by the Ministry of Health, did not alter the antioxidant activity or the LP, which can contribute to the prevention or reduction of the development of pathologies associated with SO in the newborn and promote the protection of nutrients important to him. An evaluation with other storage variables and other types of pasteurization is necessary, since the benefits detected in this study are very promising with regard to the increasing recommendation of the use of pasteurized or raw milk to replace the commonly used milk formulas and that do not have this specific benefit, in addition to others that are not described in this article.
